# Spatial distribution of SARS-CoV-2 incidence, social inequality, housing conditions, and density in South-Eastern France: keys for future epidemics

**DOI:** 10.3389/fpubh.2024.1422112

**Published:** 2024-12-06

**Authors:** Eugènia Mariné Barjoan, Bernard Prouvost-Keller, Amel Chaarana, Julie Festraëts, Carole Geloen, Kevin Legueult, Christian Pradier

**Affiliations:** ^1^Université Côte d’Azur, Centre Hospitalier Universitaire de Nice, Public Health Department, Nice, France; ^2^Université Côte d’Azur, UR2CA, Centre Hospitalier Universitaire de Nice, Nice, France

**Keywords:** SARS-CoV-2, incidence, management strategies, social inequality, overcrowded households, population density, spatial survey

## Abstract

**Introduction:**

Early in 2021, the SARS-CoV-2 incidence rate was higher in the East than in the West of the Alpes-Maritimes district in France. What was the impact of social deprivation, household overcrowding and population density per km^2^ on this difference in incidence rate?

**Methods:**

Cases were defined as persons with a first SARS-CoV-2 positive test detected between 04/01/2021 and 14/02/2021. We studied the « French Deprivation index » (FDep), rate of overcrowded households and population density/km^2^. These indicators were compared between East and West and a Standard Incidence Ratio (SIR) and an Incidence Rate Ratio (IRR) were calculated for each indicator. The link between the incidence rate and the socio-economic variables per census blocks (IRIS) was analyzed with a GLM model. Lastly, a stepwise method was used to determine the East/West incidence thresholds for which an association was observed between the incidence rate and these three indicators.

**Results:**

Among the 473 census blocks, 25,400 cases were geolocated among whom 23,867 not residing in nursing homes nor in long-term communal accommodation. Census blocks in the East included more overcrowded households (*p* = 0.009) and a higher population density (*p* < 0.001). In this area, the SARS-CoV-2 incidence was significantly higher in the most deprived census blocks (IRR = 1.614; 95%CI [1.530–1.703]), with a higher rate of overcrowded households (IRR = 1.583; 95%CI [1.508–1.663]) and higher population density (IRR = 1.062; 95%CI [1.023–1.102]). No difference was observed in the West. According to the GLM, in the East, the incidence rate was associated with the FDep index only, while no association was observed in the West. In the East, the association with FDep appeared for an incidence threshold of 210/100,000, while no threshold was identified in the West. Rates of overcrowded households were 310 vs. 370 and population density rates were 260 vs. 400 in the Eastern and Western areas, respectively.

**Conclusion:**

Our results demonstrate the benefits of conducting a spatial analysis of socio-demographic and medical data. At the start of an emerging infectious agent-related epidemic, while surveillance is not yet operational, initial prevention measures could prioritize targeting populations according to their socio-demographic characteristics.

## Introduction

**Figure 1:**
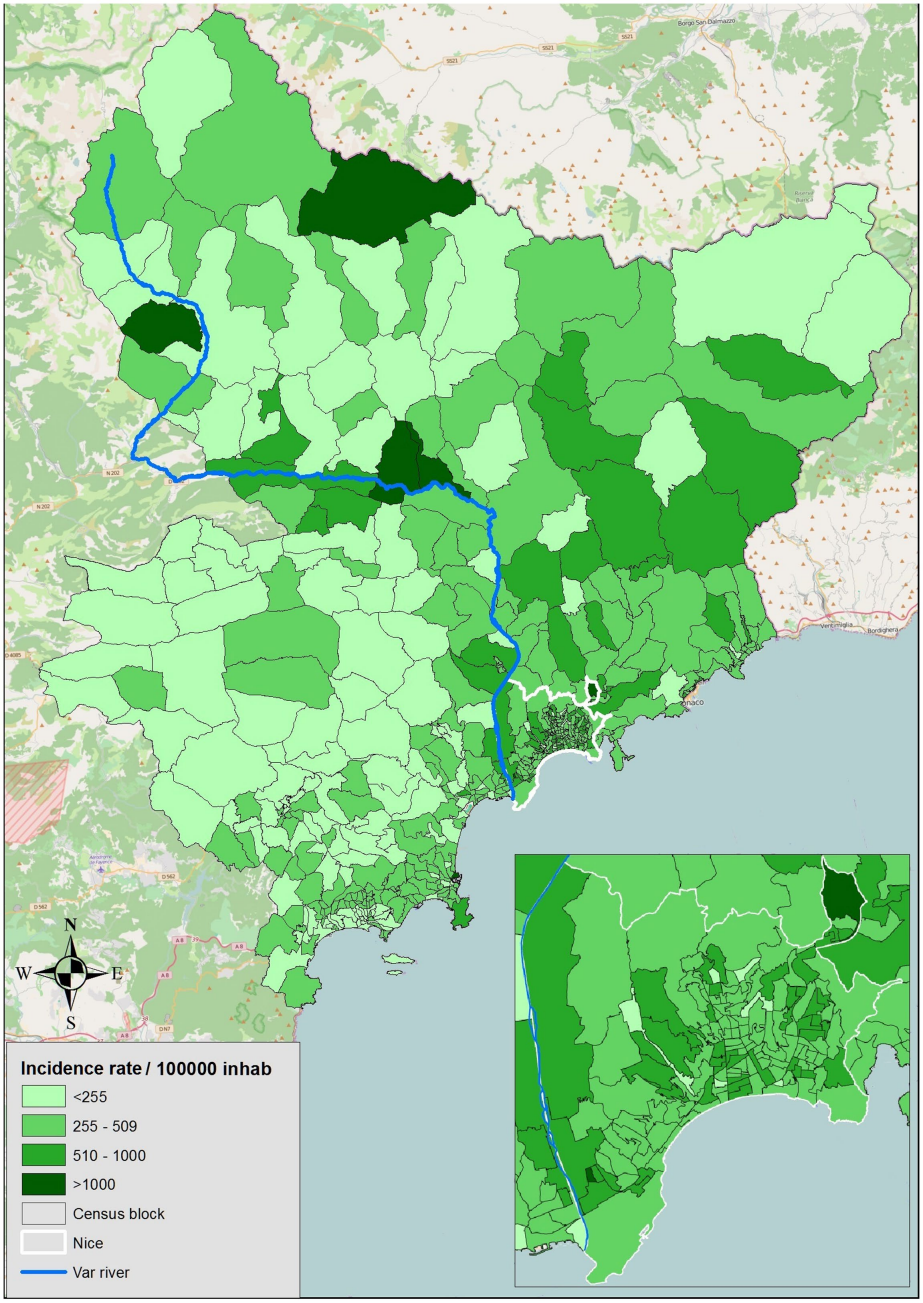
Incidence by census block in the Alpes-Maritimes district.

Early in 2021, the incidence rate of SARS-CoV-2 in the Alpes-Maritimes (AM) department was the highest in metropolitan France, increasing from 456 to 577 positive tests per 100,000 inhabitants between the first and sixth week of that year (1). On December 18^th^, 2020, it had already passed the national alert threshold of 250 cases per 100,000 inhabitants set by the prime minister as announced in a press conference of September 23^rd^, 2020. This situation led to the implementation of specific measures in the Alpes-Maritimes to limit social interactions, i.e., a curfew from 6 pm to 6 am and, as from February 26, 2021, a lockdown in the coastal area from Fridays 6 pm to Mondays 6 am (2). A spatial survey of the SARS-CoV-2 incidence rates in the AM department revealed a wide disparity, with a positive gradient running from the West to the East of the department (3) (Figure 1).

Socio-economic factors have always played a major role in epidemic dynamics (4), even in affluent countries (5, 6). Several studies have focused on their link with mortality and with the progression of the SARS-CoV-2 epidemic in Europe and worldwide (7–13). As elsewhere, results of the first study on SARS-CoV-2 and social inequality highlighted the major role of social disadvantage on the incidence of the disease (8), and these results are supported by our recent study conducted in the town of Nice (14). Further, the type of urbanisation, architecture and living conditions have been shown to be linked to infectious disease transmission (6). Household crowding has been described as directly associated with an increase in the incidence of tuberculosis (15), a finding that has led to behavioural changes aimed at reducing infectious disease transmission (6).

According to the housing survey carried out by the Observatory of inequalities (*Observatoire des inégalités*) between 2006 and 2013, the rate of overcrowded households in France rose from 24.3 to 30.5% for the poorest 10% of the population, and from 16.3 to 18.2% for the poorest 10 to 20% of households (16). In 2018, the rate of main residence overcrowding in the Alpes-Maritimes was 10.3% (53,030 overcrowded dwellings among 512,563 main residences) (17).

Studies conducted in the USA and in France in 2020 during the first wave of the epidemic suggest overcrowded dwellings could play a part in the progression of SARS-CoV-2 incidence (9–13).

Thus, in the event of SARS-CoV-2 contamination of a member of a household, the close contacts induced by overcrowding, combined with the difficulty of implementing isolation and distancing measures within the household, can contribute to the contamination of all household members (18–20) Lastly, several studies have reported an association between population density and SARS-CoV-2 incidence (4, 9, 10, 13, 21–23).

Our aim was to identify the census blocks for which social disadvantage, rate of overcrowded main residences and population density may have played a role in the variations in incidence rate of SARS-CoV-2 cases observed during the first 6 weeks of 2021 between the Western and Eastern areas of the Alpes Maritimes department.

## Methods

The study included residents of Nice with a first positive SARS-CoV-2 PCR or antigenic test result obtained between January 4, 2021, and February 14, 2021. Data were provided by the National information system for COVID 19 screening (*Système d’information national de suivi du dépistage de la COVID-19: SI-DEP*) which recorded all positive SARS-CoV-2 tests in France (24).

Variables of interest included age, date of first positive test, and residence coordinates. The address of each positive case for SARS-CoV-2 was entered in a Geographic Information System (ARCGIS 10®).

Each year, the French National Institute of Statistics and Economic Studies (*Institut national de la statistique et des études économiques*: *INSEE*) publishes socio-economic data grouped by census blocks (*Ilots Regroupés pour l’Information Statistique*: IRIS). A census block is the smallest geographical unit for which INSEE data are available and covers a relatively uniform geographic area with regard to socio-economic characteristics. Also published yearly by INSEE are 349 socio-economic indicators grouped by census blocks, 92 of which relate to housing and 28 to income. Among these indicators, we chose to consider those related to housing conditions and social disadvantage.

At the time of this study, the most recently available socio-economic data per age group had been collected in 2018. At the time, the AM department was divided into 500 census blocks, 244 were located to the West of the Var river, one of which was uninhabited (a race course), and 256 to the East, where the town of Nice is located.

For each census block, we computed three socio-economic indicators: the « French deprivation index » (FDep) score, the percentage of overcrowded main residences and the population density per km^2^. The FDep index was developed by the Surveillance centre for medical causes of death (*Centre d’épidémiologie sur les causes médicales de Décès: CépiDc*), which conducts statistical analyses of deaths in France. It is a composite indicator of social disadvantage which provides a synthetic view of social inequalities (25) based on several socio-economic data: median yearly income per consumption unit in the household, percentage of workers among the active population, percentage of graduates among those over 14 years of age, and unemployment rate. The FDep score was computed for 472 inhabited census blocks where the median income was known; this was unavailable for the 27 remaining census blocks, with a population of 2,453, i.e., 0.2% of the population of the Alpes-Maritimes. The resulting FDep scores for each census blocks were then grouped into classes using Jenk’s classification method, based on their similarities and differences, ranging from the least deprived (lowest scores) to the most deprived (highest scores), with the following score ranges: FDep1[−3.22/−1.29], the least deprived, FDep2[−1.28/−0.30], FDep3[−0.29/0.55], FDep4[0.56/1.74], FDep5[1.75/3.05] the most deprived (26).

The INSEE defines an overcrowded dwelling as one lacking at least one room, given the number of household members. The rate of overcrowded main residences in each census blocks was computed as the ratio of the number of overcrowded rooms in main residences to the number of main residences (27); we created a categorical variable divided into 6 classes based on the median rate (<1%; 1–4%; 5–9%; 10–14%; 15–19%; >19%).

The population density per census block was divided into quartiles.

The SARS-CoV-2 incidence rate was calculated per week and over the 6-week study period for each census block, per East/West geographical location, and for the entire department, and according to each of the three socio-demographic variables. Residents of nursing homes or in long-term communal accommodation were not included in the computation of the incidence rate as they are not part of the INSEE population data, but considered separately (28).

We compared the three indicators according to the Eastern and Western geographical areas. Univariate analysis was performed using the chi-square test for qualitative variables and the Mann–Whitney U-test for quantitative variables. Logistic regression was applied to identify differentiating factors between the two areas.

To check for a potential excess number of cases in relation to each of the three indicators over the whole department and in the Eastern and Western areas, we calculated the Standardized Incidence Ratio (SIR) and the Incidence Rate Ratio (IRR) with their 95% confidence intervals. We compared the most deprived population category (FDep5), the most crowded (>19%) and the quartile with the highest population density to the other categories.

A Generalized Linear Model (GLM) was used to study the link between the dependent variable (Incidence rate) and the independent socio-economic variables, per census block.

For each of the three variables, and for each geographical area (East/West), we tested the incidence rate using a crude rate stepwise approach. This allowed us to identify a threshold above which we found a significant association with each of the three variables. We chose statistical tests (Student’s T-test or Wilcoxon’s test) applicable to the type of distribution of the variable of concern.

A *p*-value <0.05 was considered significant. Data were analyzed with SPSS® and SAS® software packages.

## Results

The population count was 570,017 inhabitants in the Western area (52.5%) and 515,879 (47.5%) in the Eastern area of the Alpes-Maritimes department.

The median population count per census block was 2,198 [1,511–2,907].

**Figure 2:**
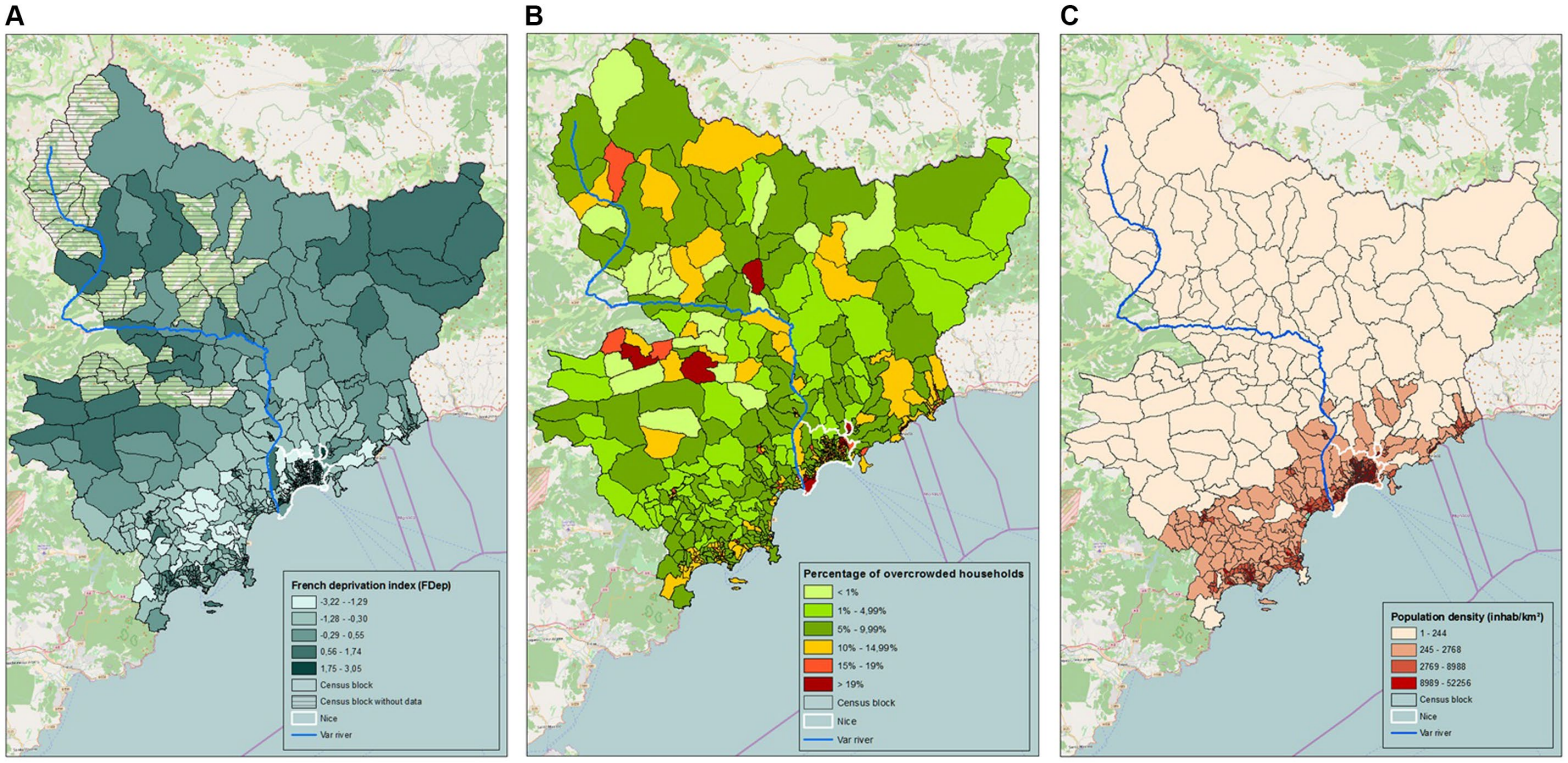
Distribution of indicators by census block. (A) French deprivation index. (B) Percentage of overcrowded households. (C) Population density.

Figure 2 shows the distribution per census block of each of the three indicators of interest: the social deprivation index (FDep), the rate of main residence overcrowding and population density. Table 1 shows the distribution of census blocks according to the indicators for West and East.

**Table 1:** Distribution of indicators according to Western and Eastern areas.

	WEST			EAST		
	Census block	Incidence		Census Block	Incidence	
	*N* = 243	Cases *N* = 9,917	Rate/100,000	*N* = 256	Cases *N* = 13,950	Rate/100,000
FDEP categories						
1	22	1,052	275	22	1,244	374
2	84	3,892	283	45	2,713	431
3	79	3,105	297	101	5,156	441
4	44	1740	300	56	3,161	442
5	3	116	333	16	1,662	696
Unknown		12			14	
Overcrowded households						
<1%	10	33	416	10	33	338
1–4%	41	1,389	248	20	912	447
5–9%	107	4,973	299	89	4,161	427
10–14%	68	2,854	293	82	4,847	428
15–19%	14	633	318	33	1913	409
>19%	3	35	215	22	2084	674
Density						
1^th^ Quartile	56	668	260	69	1,485	430
2^th^ Quartile	86	4,088	276	39	2,888	447
3^th^ Quartile	75	3,837	311	50	3,014	434
4^th^ Quartile	26	1,324	294	98	6,563	465

The median values for each of these three indicators differed significantly between East and West (Supplemental material 1). In the Eastern area, 16 census blocks (7%) harbored the most deprived population category (FDep5), versus 3 (1%) in the Western area (*p* < 0.001). Similarly, in the East, 22 census blocks (9%) contained over 19% overcrowded main residences versus 3 census blocks in the West (*p* < 0.001). Lastly, we found 98 census blocks (38%) contained the highest density quartile (over 8,988 inhabitants per square km) in the Eastern area versus 26 (11%) in the Western part (*p* < 0.001) Table 1; Supplemental material 2.

Multivariate analysis revealed significantly higher density rates (*p* < 0.001) and a higher percentage of overcrowded main residences (*p* = 0.009) in the East; we found no statistically significant difference in terms of FDep score per IRIS unit between the two geographical areas (*p* = 0.599) Supplemental material 3.

**Figure 3:**
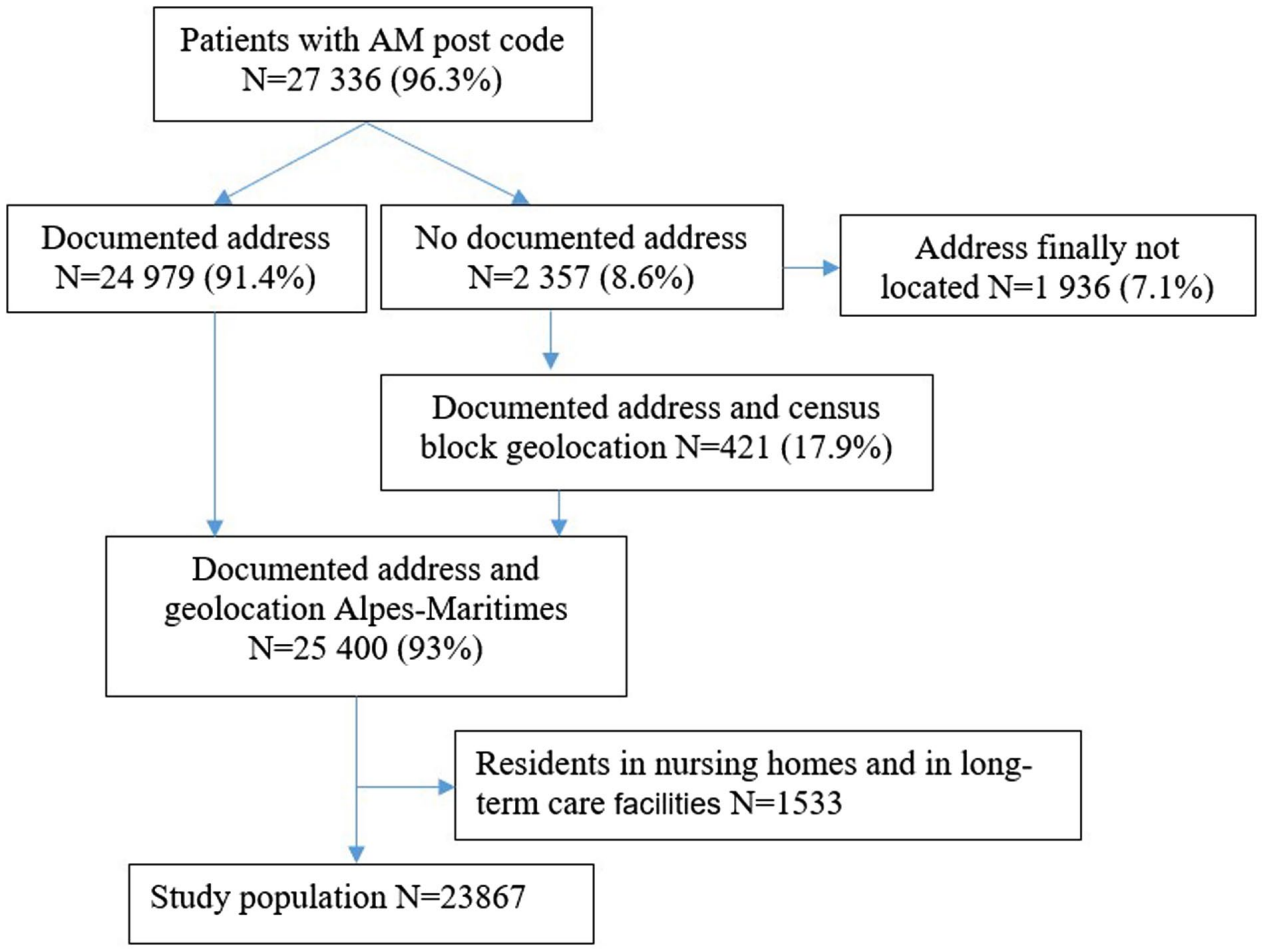
Flow chart.

We counted 27,336 SARS-CoV-2 cases in the Alpes-Maritimes over the 6-week period, with 2,357 cases with a missing address. Among them, 421 were geo-located based on the census block of their municipality. After excluding the 1,533 cases residing in nursing homes or long-term care facilities, our study population focused on 23,867 cases (Figure 3).

**Figure 4:**
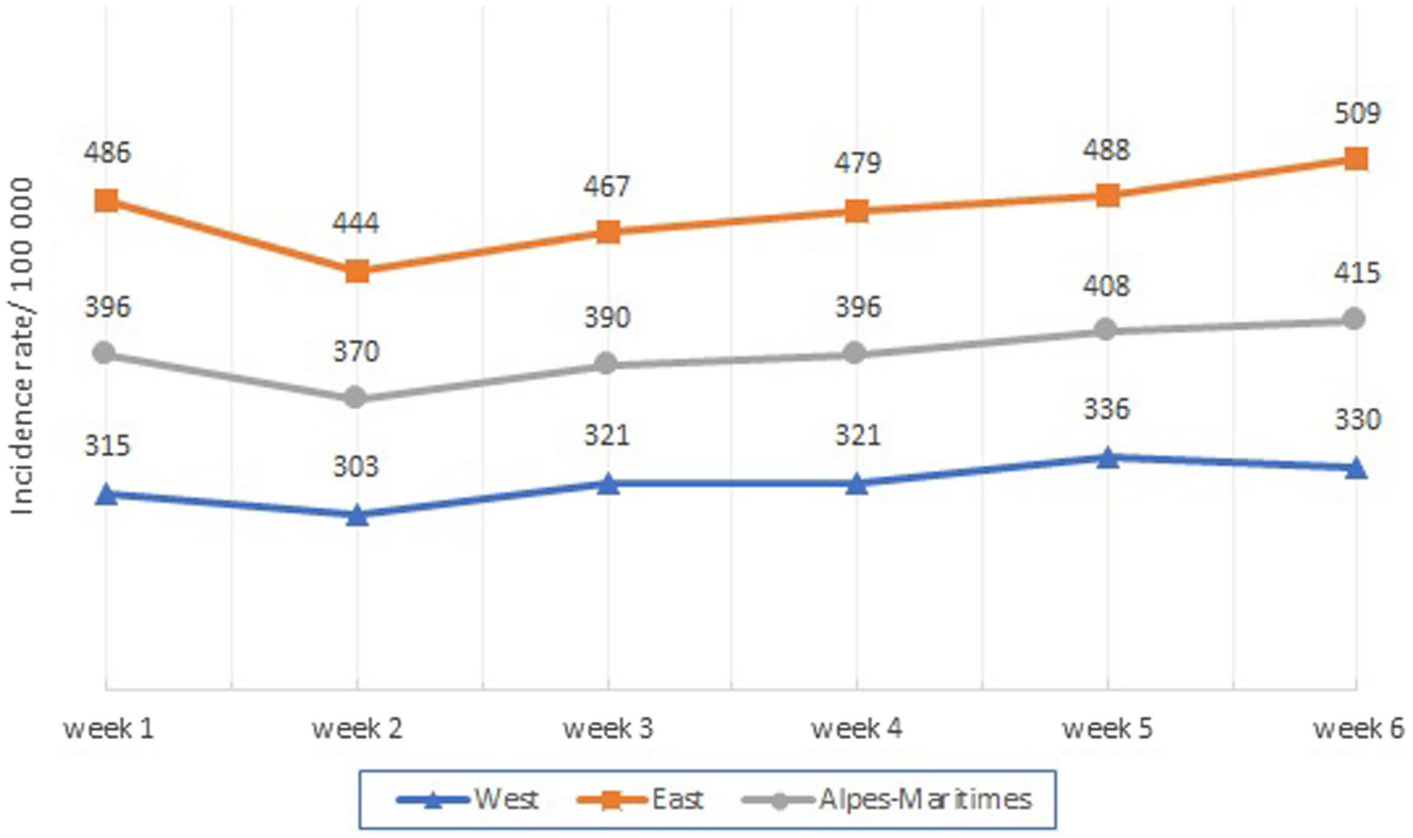
Weekly Incidence by area.

The weekly incidence rate in the AM increased from 396 to 415 cases per 100,000 inhabitants between the first and sixth week of 2021 (Figure 4).

We observed an excess number of cases in the Eastern area (SIR = 1.48 [1.45–1.50]) with a consistently higher weekly rate than in the Western area IRR = 1.49 [1.45–1.53]; Supplemental material 4.

**Figure 5:**
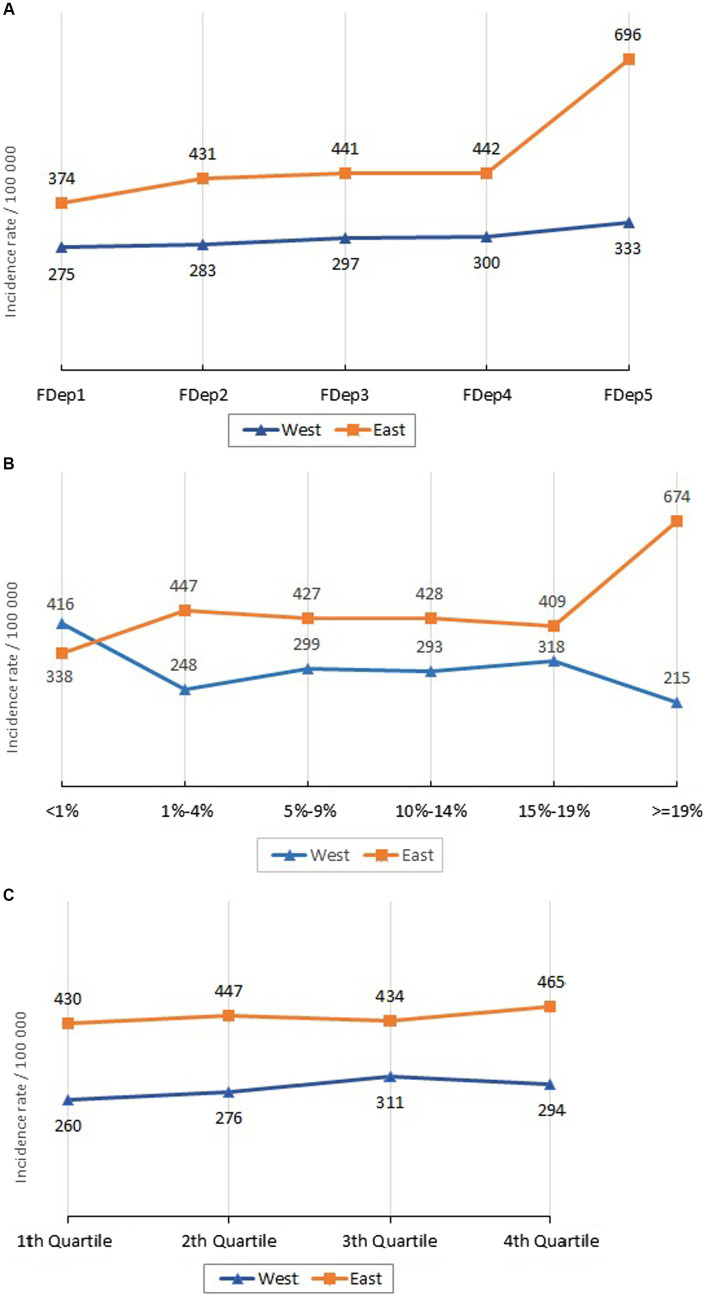
Incidence by indicator category and area. (A) Incidence by FDEP and area. (B) Incidence by overcrowded households and area. (C) Incidence by density and area.

In the AM department, the mean incidence rate was significantly higher in the census blocks harboring the most deprived population category (FDep5), where the rate of household overcrowding was above 19% and within the highest quartile for population density (Figure 5).

In the East, the incidence rate per 100,000 inhabitants was significantly higher among the FDep5 category compared to FDep1-4 (IRR = 1.614; 95%CI [1.530–1.703]), among overcrowded main residences as opposed to the others (IRR = 1,583; 95%CI [1.508–1.663]), and among the highest population density quartile (IRR = 1.062; 95%CI [1.023–1.102]). In the Western part of the AM department, no statistically significant difference was found (Table 2).

**Table 2:** Comparison of most negative indicators according to area.

	Observed cases	Expected cases	SIR	95% CI	Reference incidence rate	Incidence rate	IRR	95% CI
Most deprived vs. other categories								
East	1,662	1,030	1.61	[1.54; 1.69]^*^	696	431	1.61	[1.53; 1.70]^*^
West	116	101	1.15	[0.95; 1.38]	333	289	1.15	[0.95; 1.40]
Overcrowded household >19%								
vs. other categories								
East	2084	1,325	1.57	[1.51; 1.64]^*^	674	426	1.58	[1.51; 1.66]^*^
West	35	48	0.72	[0.50; 1.01]	215	289	0.74	[0.52; 1.06]
Density 4^th^ quartile vs. other categories								
East	6,563	6,271	1.05	[1.02; 1.07]^*^	465	438	1.06	[1.02; 1.10]^*^
West	1,322	1,304	1.01	[0.96; 1.07]	293	288	1.02	[0.95; 1.09]

* Statistically significant.

In the Eastern AM, FDep accounted for epidemic spread once the incidence rate was higher than 210 per 100,000, while in the Western part there was no statistically significant threshold value associated with the spread of SARS-CoV-2. The rate of overcrowded main residences played a role when the SARS-CoV-2 incidence rate rose above 310 in the Eastern and 370 in the Western areas. Lastly, population density was associated with an increased incidence rate when this was above 260 cases in the East and 400 cases in the West (Supplemental material 5).

The SARS-CoV-2 incidence rate as analyzed with a Generalized Linear Model was solely associated with the FDep indicator in the Eastern area, while in the Western area, no association was found with any of the three indicators (Table 3).

**Table 3:** Generalized linear model.

	EAST		WEST	
	Estimate	p-value	Estimate	p-value
Social level class (Fdep)	49.73	<=0.01*	9.132	0.572
Overcrowded households	259.411	0.357	−410	0.33
Density	−0.001	0.261	0.003	0.329

## Discussion

Our study shows that in a territory of 1 million inhabitants (the Alpes-Maritimes department in France), the spread of the SARS-CoV-2 epidemic was not geographically uniform throughout the area. Social deprivation has been identified as a major player in epidemic dynamics (8, 13, 14, 20, 29). The present study suggests that this indicator varies according to the geographical area and that it could be associated with an incidence threshold. These results are in favor of analysing epidemic dynamics such as that of SARS-CoV-2 by combining medical and socio-demographic data, integrated within a Geographical Information System (GIS) (30).

Several studies have analyzed the role of socio-demographic indicators on the incidence of SARS-CoV-2 at the national, regional (8, 10), or municipal level (31–35). Studies focusing on incidence rate at the neighborhood level within a municipality have highlighted its variability in space (21, 29, 34) and time (35, 36).

We considered the FDep social deprivation index as the most suitable to measure social inequality, as it was specifically developed and validated for the French population. The FDep distribution into five classes using Jenk’s algorithm was more in line with our objective, i.e., to identifiy geographically-specified population groups, as this method limits within-class variation while maximizing inter-class variation. This method does not classify data in an arbitrary fashion, subdividing the population in groups of 20% as with quintiles or 10% as with deciles, but according to their uniformity, with thresholds that we feel are more in line with reality. Regardless of the variables or indicators chosen to characterize social deprivation, studies conducted in France (8, 20), Britain (37), Switzerland (38), Germany (39) or Spain (35, 40) all reported that it was systematically associated with a higher incidence rate of SARS-CoV-2 and to less frequent testing (8, 41).

Overcrowding is variably defined in different studies. Two French studies defined overcrowded housing as a situation where at least two persons live in a dwelling with less than 18m^2^ per person (8, 20). In line with the INSEE and other studies, we defined it as a situation where there is more than one person per room (12, 27, 42). The link between household overcrowding and incidence rate has been reported in the USA, independently of situations where three generations or more live within the same household (20).

In studies focusing on urban populations, the rate of overcrowded housing was found to be associated with Covid-19-related morbidity (12, 21) or mortality (7, 42).

In terms of population density, there was either no definition or that of a concentration of individuals at a given time within a specific setting (airports, travel or commercial centers) (43, 44), or the number of inhabitants per square km, as in our study (45). In several studies, population density is expressed quantitatively or in categories with variable thresholds (8, 10, 21, 22, 34, 35, 37).

In the European Union, a high population density is defined as a situation where >50% of the population live in an area where the density is ≥1,500 inhabitants per km^2^. In the USA, Lee et al. reported a low population density when more than 50% of residents lived in a county of fewer than 1,000 inhabitants per km^2^, a medium population density between 1,000 and 5,000 inhabitants per km^2^ and a high density when there were more than 5,000 per km^2^ (10). In Great Britain, density was studied according to urban and rural classes (37), or in quartiles (22). Despite such disparities in definition and classification, some studies have reported an association between population density and viral transmission (8, 10, 22, 35). Our density analysis by quartiles was linked to an excess number of cases limited to the East of the Alpes-Maritimes department. This is likely to be related to the presence of a large municipality, i.e., Nice, which is home to 66% of the AM population (10, 14, 21) Population density in urban settings has been linked to a higher rate of viral transmission (23, 24, 35, 45).

The Alpes-Maritimes coastline harbors 95.6% of the population in this department (46). There is a major degree of shuttling between East and West but, as reported in another publication, viral circulation appears to have been more influenced by socio-demographic factors than by population mobility (35).

We found no publication reporting an incidence threshold for the three studied variables that would point to their impact on viral spread. We found a link between these variables and the incidence rate in the Eastern but not in the Western part of the department. This may be linked to the low number of census blocks with unfavourable characteristics in the Western area regarding the three studied indicators.

By actively searching for addresses and checking their consistency, we were able to study 93% of cases, while the remaining 7% could not be allocated to a census block, as it was not possible to associate them with the indicators provided by the INSEE. This may have partly biased our results. However, in a French study that used the same source for SARS-CoV-2 cases (SI-DEP), 20.5% lacked an accurate address; to compensate for this, cases were assigned to census blocks on a probability basis (8).

Although the study was conducted in 2021, we chose to use the 2018 INSEE data, which was the most recent year for which distribution per age group and census block was available, as well as the related socio-economic data. The delay between the study period and the one for which socio-economic data are available has already been mentioned in other French studies (8). We thus considered that this bias did not compromise the validity of our results, despite potential minor changes in socio-economic data over the 4-year period.

The missing data for certain variables, such as the poverty index in 27 of the 499 inhabited census blocks, could have biased our correlation results. This concerned 2,453 inhabitants (0.2% of the AM population). However, we considered that these missing data had little impact on our results.

The use of aggregated data for socio-demographic indicators offers the advantage of facilitating their collection, while individual data are difficult to obtain in this type of survey (21).

The limited 6-week study period was chosen following the publication of comprehensive incidence data from the Alpes-Maritimes area, when these data were validated and before a possible impact of vaccination which began gradually as from February 25, 2021.

However, we were able to show that during an overall epidemic peak in our area, there were two completely different trends in incidence rates between the Eastern and Western districts of the Alpes-Maritimes, despite significant population mobility.

During the study period, a curfew had been introduced between 6 pm and 6 am throughout the department. Given the results of our analysis regarding census blocks, it appears more relevant to prioritize the implementation of preventive measures and management in those territories where socio-demographic factors favor epidemic spread, rather than to approach the situation in terms of administrative districts.

## Conclusion

Based on census blocks, this study led us to identify areas with higher incidence rates related to social deprivation, housing conditions and population density. Our results thus demonstrate the benefits of conducting a spatial analysis of socio-demographic and medical data.

At the start of an emerging infectious agent-related epidemic, with an exponentially increasing incidence rate, unknown risk factors for severe illness and surveillance not yet operational, prevention and screening measures could prioritize targeting populations previously identified on the sole basis of their socio-demographic characteristics. Monitoring the incidence rate would thus become a medium- and long-term evaluation and surveillance tool. This approach would make it possible to adapt prevention measures rapidly in future epidemics.

## Data Availability

The raw data supporting the conclusions of this article will be made available by the authors, without undue reservation.
