# Supplementary material for: Spatial distribution of SARS-CoV-2 incidence, social inequality, housing conditions, and density in South-Eastern France: keys for future epidemics
Source: Front Public Health. 2024 Dec 6;12:1422112. doi: 10.3389/fpubh.2024.1422112 (PMC11659207; doi:10.3389/fpubh.2024.1422112)
Supplement: Supplementary file 1 [file Supplementary_file_1.docx]

Suppl. mat_1_Comparison of median values for the three indicators between Eastern and Western areas

| EAST | | WEST | | p |  |  |
| --- | --- | --- | --- | --- | --- | --- |
| FDep | 0.2067954 [-0.3471066 ; 0.6752922] | | -0.2286100 [-0.8100779 ; 0.4113172] | | <0.001* | |
| Overcrowded households | 10.4% [7.2% ; 14.1%] | | 8.1% [5.4% ; 11.0%] | | <0.001* | |
| Density | 5 405 [190 ; 15 148] | | 1 566 [263 ; 5855] | | <0.001* | |
| * statiscally significant |  | |  | |  | |

Suppl. mat_2_Distribution of census block by indicators according East and West

Image1. FDep


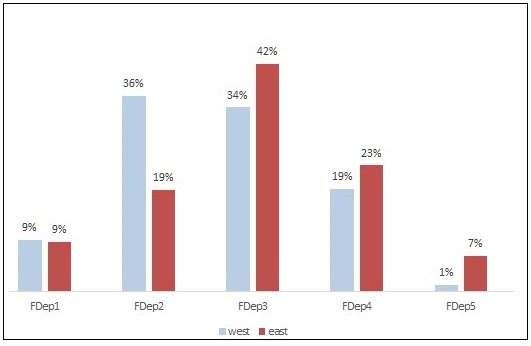


Image 2. Overcrowded households


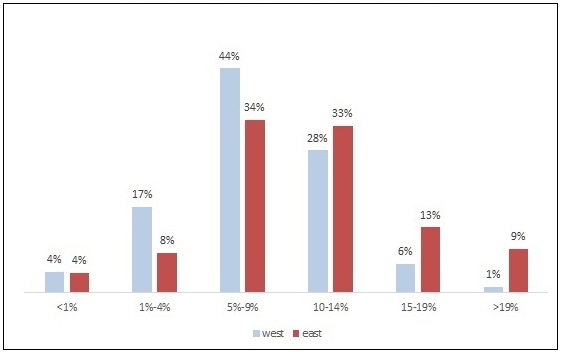


Image 3. Density


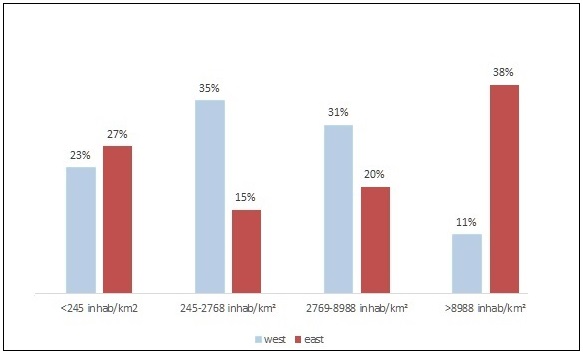


Supl mat_3_Regression analysis East West

|  |  |  |  |  |  |  |  |
| --- | --- | --- | --- | --- | --- | --- | --- |
|  | B | E.S | Wald | p-value | Exp(B) | 95% CI | |
| Density | 0.000 | 0.000 | 19.399 | 0.000 * | 1.000 | [1.00004 ; 1.00011] | |
| FDEP | -0.069 | 0.132 | 0.276 | 0.599 | 0.933 | [0.72108 ; 1.20778] | |
| Overcrowded households | 0.073 | 0.028 | 6.862 | 0.009 * | 1.076 | [1.01866 ; 1.13699] | |

* statistically significant

Suppl. mat_4_IRR et SIR East West

| West Observed cases | East Observed cases | East expected cases | SIR | 95%CI | West Incidence rate | East Incidence rate | IRR | 95%CI |
| --- | --- | --- | --- | --- | --- | --- | --- | --- |
| 10978 | 14819 | 10036 | 1.48 | [1.45 ; 1.50] | 321 | 479 | 1.49 | [1.45 ; 1.53] |

Suppl. mat_5_ Threshold incidence indicators East West

| FDep | | | | Overcrowded Households | | | | | | | Density | | | | | |
| --- | --- | --- | --- | --- | --- | --- | --- | --- | --- | --- | --- | --- | --- | --- | --- | --- |
| EAST | | WEST | | EAST | | WEST | | | | EAST | | | | WEST | |  |
| IR | p-value | IR | p-value | IR | p-value | | IR | p-value | IR | | | p-value | IR | | p-value |  |
| 100 | 0.1 | 40 | 0.1 | 200 | 0.3 | | 250 | 0.3 | 150 | | | 0.3 | 200 | | 0.2 |  |
| 150 | 0.4 | 100 | 0.4 | 250 | 0.1 | | 300 | 0.1 | 200 | | | 0.1 | 300 | | 0.1 |  |
| 190 | 0.09 | 150 | 0.4 | 300 | 0.07 | | 350 | 0.08 | 250 | | | 0.1 | 350 | | 0.09 |  |
| 200 | 0.06 | 200 | 0 .6 | 305 | 0.06 | | 365 | 0.06 | 255 | | | 0.06 | 390 | | 0.06 |  |
| 210 | 0.05* | 250 | 0.6 | 310 | 0.05* | | 370 | 0.05* | 260 | | | 0.05* | 400 | | 0.05* |  |
| 215 | 0.05* | 300 | 0.5 | 315 | 0.04* | | 275 | 0.04* | 265 | | | 0.04* | 410 | | 0.04* |  |
| 220 | 0.04* | 350 | 0.2 | 350 | 0.01* | | 285 | 0.03* | 270 | | | 0.03* | 450 | | 0.04* |  |
| 260 | 0.04* | 400 | 0.8 |  |  | |  |  |  | | |  |  | |  |  |
| 270 | 0.02* | 500 | 0.9 |  |  | |  |  |  | | |  |  | |  |  |
|  |  | 550 | 0.9 |  |  | |  |  |  | | |  |  | |  |  |
|  |  | 600 | 0.9 |  |  | |  |  |  | | |  |  | |  |  |
|  |  | 650 | 0.9 |  |  | |  |  |  | | |  |  | |  |  |
|  |  | 700 | 0.9 |  |  | |  |  |  | | |  |  | |  |  |
|  |  | <>950 | 0.9 |  |  | |  |  |  | | |  |  | |  |  |

IR= Incidence rate

* statiscally significant
